# PSMA PET/CT: joint EANM procedure guideline/SNMMI procedure standard for prostate cancer imaging 2.0

**DOI:** 10.1007/s00259-022-06089-w

**Published:** 2023-01-05

**Authors:** Wolfgang P. Fendler, Matthias Eiber, Mohsen Beheshti, Jamshed Bomanji, Jeremie Calais, Francesco Ceci, Steve Y. Cho, Stefano Fanti, Frederik L. Giesel, Karolien Goffin, Uwe Haberkorn, Heather Jacene, Phillip J. Koo, Klaus Kopka, Bernd J. Krause, Liza Lindenberg, Charles Marcus, Felix M. Mottaghy, Daniela E. Oprea-Lager, Joseph R. Osborne, Morand Piert, Steven P. Rowe, Heiko Schöder, Simon Wan, Hans-Jürgen Wester, Thomas A. Hope, Ken Herrmann

**Affiliations:** 1 Department of Nuclear Medicine, University of Duisburg-Essen and German Cancer Consortium (DKTK)-University Hospital Essen, Hufelandstraße 55, 45147 Essen, Germany; 2 PET Committee of the German Society of Nuclear Medicine, Marburg, Germany; 3 Department of Nuclear Medicine, Klinikum Rechts Der Isar, Technical University of Munich, Munich, Germany; 4 Division of Molecular Imaging & Theranostics, Department of Nuclear Medicine, University Hospital Salzburg, Paracelsus Medical University, Salzburg, Austria; 5 Institute of Nuclear Medicine, UCLH NHS Foundation Trust, London, UK; 6 Ahmanson Translational Theranostics Division, Department of Molecular and Medical Pharmacology, University of California Los Angeles, Los Angeles, CA USA; 7 Division of Nuclear Medicine and Theranostics, IEO European Institute of Oncology, IRCCS, Milan, Italy; 8 Department of Oncology and Hemato-Oncology, University of Milan, Milan, Italy; 9 Department of Radiology, School of Medicine and Public Health, University of Wisconsin, Madison, WI USA; 10 IRCCS, AOU Bologna, Bologna, Italy; 11 Department of Nuclear Medicine, University Hospital Düsseldorf, Medical Faculty, Heinrich-Heine-University and Department of Nuclear Medicine, University Hospital Heidelberg, Heidelberg, Germany; 12 Department of Nuclear Medicine, Division of Nuclear Medicine and Molecular Imaging, University Hospital Leuven, KU Leuven, Louvain, Belgium; 13 Department of Nuclear Medicine, University Hospital Heidelberg, Heidelberg, Germany; 14 Dana-Farber Cancer Institute/Brigham and Women’s Hospital, Boston, USA; 15 Banner MD Anderson Cancer Center, Phoenix, AZ USA; 16 Institute of Radiopharmaceutical Cancer Research, Helmholtz-Zentrum Dresden-Rossendorf (HZDR), Dresden, Germany; 17 School of Science, Faculty of Chemistry and Food Chemistry, Technical University Dresden, Dresden, Germany; 18 German Cancer Consortium (DKTK), Partner Site Dresden, Dresden, Germany; 19 Department of Nuclear Medicine, University Medical Center, University of Rostock, Rostock, Germany; 20 Molecular Imaging Branch, Center for Cancer Research, National Cancer Institute, NIH, Bethesda, MD USA; 21 Division of Nuclear Medicine and Molecular Imaging, Department of Radiology, Emory University School of Medicine, Atlanta, GA USA; 22 Department of Nuclear Medicine, University Hospital RWTH Aachen University, Aachen, Germany; 23 Department of Radiology and Nuclear Medicine, Maastricht University Medical Center (MUMC+), Maastricht, The Netherlands; 24 Department of Radiology & Nuclear Medicine, Amsterdam University Medical Centers, VU University Medical Center, Cancer Center Amsterdam, De Boelelaan 1117, 1081 HV Amsterdam, The Netherlands; 25 Department of Radiology, Division of Molecular Imaging and Therapeutics, Weill Cornell Medicine, New York, NY USA; 26 Department of Radiology, Division of Nuclear Medicine and Molecular Imaging, University of Michigan, Ann Arbor, MI USA; 27 Division of Nuclear Medicine and Molecular Imaging, The Russell H. Morgan Department of Radiology and Radiological Science, Johns Hopkins University School of Medicine, Baltimore, MD USA; 28 Memorial Sloan-Kettering Cancer Center, New York, NY USA; 29 Pharmaceutical Radiochemistry, Technische Universität München, Walther-Meißner-Str. 3, 85748 Garching, Germany; 30 Department of Radiology and Biomedical Imaging, University of California, San Francisco, CA USA

**Keywords:** PSMA, PET, Prostate cancer, Staging, Restaging, Guideline

## Abstract

Here we aim to provide updated guidance and standards for the indication, acquisition, and interpretation of PSMA PET/CT for prostate cancer imaging. Procedures and characteristics are reported for a variety of available PSMA small radioligands. Different scenarios for the clinical use of PSMA-ligand PET/CT are discussed. This document provides clinicians and technicians with the best available evidence, to support the implementation of PSMA PET/CT imaging in research and routine practice.

## Preamble

The Society of Nuclear Medicine and Molecular Imaging (SNMMI) is an international scientific and professional organization founded in 1954 to promote the science, technology, and practical application of nuclear medicine. The European Association of Nuclear Medicine (EANM) is a professional non-profit medical association founded in 1985 that facilitates communication worldwide between individuals pursuing clinical and research excellence in nuclear medicine. SNMMI and EANM members are physicians, technologists, and scientists specializing in the research and practice of nuclear medicine.

The SNMMI and EANM will periodically define new guidelines for nuclear medicine practice to help advance the science of nuclear medicine and to improve the quality of service to patients throughout the world. Existing practice guidelines will be reviewed for revision or renewal, as appropriate, on their fifth anniversary or sooner, if indicated.

Each practice guideline, representing a policy statement by the SNMMI/EANM, has undergone a thorough consensus process in which it has been subjected to extensive review. The SNMMI and EANM recognize that the safe and effective use of diagnostic nuclear medicine imaging requires specific training, skills, and techniques, as described in each document. Reproduction or modification of the published practice guideline by those entities not providing these services is not authorized.

These guidelines are an educational tool designed to assist practitioners in providing appropriate care for patients. They are not inflexible rules or requirements of practice and are not intended, nor should they be used, to establish a legal standard of care. For these reasons and those set forth below, both the SNMMI and the EANM caution against the use of these guidelines in litigation in which the clinical decisions of a practitioner are called into question.

The ultimate judgment regarding the propriety of any specific procedure or course of action must be made by the nuclear medicine physician or medical physicist in light of all the circumstances presented. Thus, there is no implication that an approach differing from the guidelines, standing alone, is below the standard of care. To the contrary, a conscientious practitioner may responsibly adopt a course of action different from that set forth in the guidelines when, in the reasonable judgment of the practitioner, such course of action is indicated by the condition of the patient, limitations of available resources, or advances in knowledge or technology subsequent to publication of the guidelines.

The practice of medicine includes both the art and the science of the prevention, diagnosis, alleviation, and treatment of disease. The variety and complexity of human conditions make it impossible to always reach the most appropriate diagnosis or to predict with certainty a particular response to treatment. Therefore, it should be recognized that adherence to these guidelines will not ensure an accurate diagnosis or a successful outcome. All that should be expected is that the practitioner will follow a reasonable course of action based on current knowledge, available resources, and the needs of the patient to deliver effective and safe medical care. The sole purpose of these guidelines is to assist practitioners in achieving this objective.

## Introduction

Prostate-specific membrane antigen (PSMA)-directed positron emission tomography/computed tomography (PET/CT) is a non-invasive diagnostic technique to image PSMA positive lesions in individuals with prostate cancer. PSMA is a transmembrane protein with an extracellular binding site. PSMA tissue expression is high on the cell surface of prostatic tissues including prostate cancer; however, despite the name, PSMA is not specific to prostate tissue. The PSMA protein can be found in low concentrations in many other organs. PSMA is also termed glutamate carboxy-peptidase II, referring to its role in neuronal glutamate synthesis in the neurochemistry context, or folate hydrolase 1 (FOLH1), referring to the encoding gene.

Increased PSMA expression is seen most notably in prostate cancer, but has also been found in the neovasculature of a variety of other malignancies [1]. Most adenocarcinomas of the prostate express high levels of PSMA in primary and metastatic lesions [2, 3]. Elevated PSMA expression in conjunction with its role in glutamate and folate metabolism may be associated with a survival advantage for tumor cells in conditions of cellular stress [4, 5]. The regulation of PSMA is complex, with the involvement of androgen receptor (AR), PI3K/Akt, and DNA damage response pathways [6]. Elevated PSMA expression was previously associated with advanced metastatic or hormone-refractory prostate cancer [7], poor disease outcome [8], and the presence of deficient DNA damage repair pathways [9].

## Definitions

The following definitions are made in accordance with Boellaard et al. [10] and Fendler et al. [11]:

PSMA-ligand: Refers to a group of ligands (here [^68^ Ga]Ga-PSMA-11, [^68^ Ga]Ga-PSMA-I&T, [^18^F]F-DCFPyL, [^18^F]F-PSMA-1007, or [^18^F]F-rhPSMA-7.3) that targets the prostate-specific membrane antigen.

PET/CT: An integrated or multimodality PET/CT system is a physical combination of PET and CT, which allows sequential acquisition of the PET and CT portions. The patient remains in the same position for both examinations.

PET/MRI: An integrated or multimodality PET/MRI system is a physical combination of PET and MRI, which allows sequential or simultaneous acquisition of the PET and MRI portions. The patient remains in the same position for both examinations. PSMA-ligand PET/MRI has been reported for several applications, including staging at initial diagnosis or biochemical recurrence (BCR). However, PET/MRI protocols are outside the scope of this guideline.

PSMA-ligand PET: A detector system that measures the three-dimensional (3D) distribution of PSMA-ligands, producing semi-quantitative images that allow non-invasive assessment of PSMA expression.

A PSMA-ligand PET/CT examination may cover various coaxial imaging ranges. These are described as follows (defined in Current Procedural Terminology 2016):

Total-body PET: From the top of the head through the feet.

Whole-body PET: From the base of the skull to mid-thigh. This range covers most of the relevant portions of the body in many oncological diseases (standard for both Europe and the USA). If indicated, cranially extended imaging may also cover the brain in the same scan (vertex to mid-thigh).

Standardized uptake value (SUV): Quantification of PSMA-ligand PET/CT is defined here as measuring relative PSMA-ligand concentrations using standardized uptake value (SUV) [12] because SUV represents the most commonly used semi-quantitative parameter for analysis of tracer uptake.

Maximum standardized uptake value (SUV_max_): SUV_max_ is defined as the SUV of the single voxel in a region of interest that presents the highest uptake on the attenuation corrected PET image.

CT applies a combined X-ray source and detector rotating around the patient to acquire tomographic data. CT generates 3D images of tissue density, which allows for attenuation correction of PET and anatomical/tumor visualization with a high spatial resolution. A PET/CT examination can include different types of CT scans depending on the CT characteristics, the radiation dose, and the use (or not) of oral and/or intravenous contrast agents:

Low-dose CT scan: A CT scan performed only for attenuation correction (CT-AC) and anatomical correlation of PET findings (with reduced voltage and/or current of the X-ray tube settings), i.e., a low-dose CT is not intended for a dedicated radiological interpretation. This scan delivers less radiation to the patient.

Diagnostic CT scan: A CT scan with or without intravenous and/or oral contrast agents, commonly using higher X-ray doses than low-dose scans for higher resolution imaging. A diagnostic CT scan should be performed according to applicable local or national protocols and guidelines.

Biochemical recurrence (BCR): Recurrence of prostate cancer due to rising PSA after definitive surgical or radiation therapy.

Biochemical persistence (BCP): Persistence of prostate cancer due to continuously elevated PSA despite surgical treatment.

Radioligand therapy (RLT): Internal radiation of prostate cancer lesions by the application of PSMA-directed therapeutic radioligands.

Non-metastatic castration-resistant prostate cancer (nmCRPC): Castration-resistant prostate cancer with no detected metastases on whole-body cross-sectional imaging (CT/MRI) and bone scan.

## PSMA-ligand PET — a novel class for prostate cancer imaging

PSMA-ligands for PET/CT imaging were first radiosynthesized and validated in preclinical models at Johns Hopkins University [13, 14]. Later, [^68^ Ga]Ga-PSMA-11, developed by the Heidelberg group [15], demonstrated high affinity to human PSMA and specific internalization into prostate cancer cells. [^68^ Ga]Ga-PSMA-11 biodistribution was shown to correspond to known cellular expression of PSMA across organs [16]. Other ^68^ Ga-PSMA-ligands ([^68^ Ga]Ga-PSMA-617, [^68^ Ga]Ga-PSMA-I&T) demonstrated similar biodistribution and imaging properties [17, 18]. During this time, several ^18^F-labelled ligands have also been developed and assessed in clinical trials [19–22].

The radiopharmaceuticals [^68^ Ga]Ga-PSMA-11, [^68^ Ga]Ga-PSMA-I&T, [^18^F]F-DCFPyL, [^18^F]F-PSMA-1007, and [^18^F]F-rhPSMA-7.3 are most advanced in the process of clinical implementation and/or regulatory approval. Most clinical evidence is based on [^68^ Ga]Ga-PSMA-11 since it has been in use the longest. There is no large head-to-head prospective study with lesion validation to directly compare the diagnostic accuracy of different PSMA-ligands. A small comparative prospective study demonstrated similar uptake in tumor lesions of [^18^F]F-DCFPyL and [^18^F]F-PSMA-1007 [23]. Radioligands differ in terms of radionuclide label, underlying radiochemistry, and associated organ biodistribution. Different physiologic distribution and image interpretation pitfalls were noted [24]. However, there is no evidence to date that one specific PSMA radioligand has superior diagnostic accuracy with improved clinical outcome compared to another. Due to their similarity, [^68^ Ga]Ga-PSMA-11, [^68^ Ga]Ga-PSMA-I&T, [^18^F]F-DCFPyL, [^18^F]F-PSMA-1007, and [^18^F]F-rhPSMA-7.3 are considered a common class of PSMA-directed small-ligand radiotracers for PET/CT and will henceforth be collectively referred to as PSMA-ligands.

## Goals

This guideline supports physicians in recommending, acquiring, interpreting, and reporting the results of PSMA-ligand PET/CT for initial diagnosis, staging, and restaging of prostate cancer. In this intent, this document reports on patient selection, PET/CT acquisition, image interpretation, and written summary of the clinical report. Specific advice is given for the most common PSMA small radioligands available and for clinical scenarios with frequent use of PET/CT, including staging, restaging, and assessment for suitability of PSMA RLT. This document provides clinicians and technicians with the best available evidence. Sections inform where robust evidence is lacking, and report data to achieve the best possible diagnostic efficacy and study quality.

Adequate precision, accuracy, and repeatability are essential for the clinical management of patients. Standardization supports the clinical implementation of PSMA-ligand PET/CT and enhances subsequent research.

## Appropriateness of use criteria

Since the introduction of PSMA-ligand PET/CT, several prospective multicenter trials have reported on the diagnostic and clinical value of PSMA-ligand PET/CT. The criteria outlined in this guideline are based on the currently available evidence. The specific use varies between institutions based on experience and availability. An overview focusing on appropriate use criteria has been recently published [25]. The most important indications are summarized in Table 1. Current evidence for these indications is reported in the following sections.

**Table 1:** Indications for PSMA-ligand PET/CT

Routine clinical use
Initial staging of prostate cancer
Localization of recurrent (BCR) or persistent (BCP) prostate cancer
Localization of prostate cancer which is non-metastatic by conventional imaging (nmCRPC)
Staging before PSMA-directed radioligand therapy
Potential clinical applications
Guidance of prostate biopsy
Imaging metastatic prostate cancer
Monitoring of systemic treatment for metastatic prostate cancer

### Initial staging of unfavorable intermediate to high-risk prostate cancer

In patients with risk features (Gleason score 4+3 / ISUP grade 3 or higher, PSA > 20 ng/mL, clinical stage T2c–3a), the likelihood of distant metastases is increased. PSMA-ligand PET imaging demonstrated higher accuracy for disease localization in individuals with newly diagnosed prostate cancer compared with conventional imaging. In the phase III multicenter randomized ProPSMA trial, [^68^ Ga]Ga-PSMA-11 PET/CT resulted in 27% greater accuracy when compared with CT and bone scan for staging of individuals with initial high-risk prostate cancer [26]. Findings were validated by histopathology, imaging, or biochemistry at a 6-month follow-up.

In two phase II/III multicenter studies, [^18^F]F-DCFPyL and [^68^ Ga]Ga-PSMA-11 PET/CT demonstrated high specificity (≥ 95%) for detection of pelvic lymph node metastases in individuals with intermediate or high-risk prostate cancer undergoing radical prostatectomy [27, 28]. However, due to low sensitivity in the 40% range, a negative PSMA PET scan cannot exclude the presence of pelvic lymph node micrometastases due to the intrinsic limitations of current PET technology. Other trials are underway to assess the impact of the inclusion of PSMA-ligand PET in clinical management pathways on patient survival [29].

Such phase III prospective level evidence underlines the value of PSMA-ligand PET for accurate disease localization and risk stratification in individuals with newly diagnosed prostate cancer and high-risk features.

### Localization of recurrent (BCR) or persistent (BCP) prostate cancer following curative-intent therapy

BCR is defined as an increase in PSA to ≥ 0.2 ng/mL, measured at 6 to 13 weeks following prostatectomy, and confirmed by a second PSA level > 0.2 ng/mL [30]. BCP is defined as persistently elevated PSA ≥ 0.1 ng/mL more than 6 weeks after prostatectomy [31].

In patients who have undergone curative-intent radiation therapy, BCR is defined as a rise in PSA of ≥ 2 ng/mL above the nadir achieved after radiotherapy with or without androgen deprivation therapy (ADT) [32]. In patients with BCR or BCP, precise tumor localization with stratification of local, locoregional, or distant disease is critical for subsequent management.

Several prospective multicenter studies reported on the accuracy of PSMA-ligand PET in these settings. [^68^ Ga]Ga-PSMA-11 and [^18^F]F-DCFPyL PET/CT demonstrated high patient- and region-level detection rates and positive predictive value for the localization of prostate cancer in the setting of BCR or BCP [33–35]. Accuracy was superior to conventional imaging [36], [^18^F]F-choline PET/CT [37], and [^18^F]F-fluciclovine PET/CT [38] in head-to-head assessments. Interobserver agreement of [^68^ Ga]Ga-PSMA-11 is high. The PSMA-ligand PET detection rate was associated with PSA level and doubling time [33, 39], Gleason score [36], and PSMA expression of the primary [40, 41]. The accuracy of PSMA-ligand PET translated into a significant impact on management in several prospective studies [42, 43]. Trials are underway to assess the impact on patient survival [44].

Current prospective evidence underlines the role of PSMA-ligand PET for prostate cancer localization at BCR or BCP and demonstrates superiority over conventional or other forms of molecular imaging.

### Localization of castrate resistant prostate cancer which is non-metastatic by conventional imaging (nmCRPC)

nmCRPC is characterized by biochemical disease progression despite sufficient ADT. This is defined by the combined occurrence of several conditions: (a) castrate serum testosterone < 50 ng/dL, (b) three consecutive rises in PSA resulting in two 50% increases above the nadir, (c) a PSA > 2 ng/mL (EAU, European Association of Urology) or a PSA > 1 ng/mL (The Prostate Cancer Working Group 3, PCWG3), and (d) lack of metastatic spread on conventional imaging [45–48].

PSMA-ligand PET/CT has been studied in the nmCRPC population [49–52]. PSMA-ligand PET/CT detects locoregional only disease in 44% and distant disease in 55% for patients with nmCRPC and risk features [49]. Thus, PSMA-ligand PET/CT detects disease extent in patients with nmCRPC (defined by conventional imaging) with high accuracy and leads to a considerable stage migration [53]. Accurate localization of disease extent by PSMA-ligand PET/CT may aid patient stratification in clinical trials and adds information for therapy guidance. However, the impact on clinical outcome has yet to be determined prospectively.

### Staging before PSMA-directed RLT for metastatic prostate cancer

PSMA-ligand PET/CT can be performed in patients with advanced prostate cancer to confirm eligibility for RLT and to assess the likelihood of response to RLT.

Documentation of PSMA expression in metastatic sites is required prior to the initiation of RLT. ^177^Lu-PSMA-617 RLT was approved by the FDA for the treatment of eligible patients with metastatic castration-resistant prostate cancer in March of 2022. The phase III VISION trial demonstrated improved radiographic progression-free survival (8.7 vs. 3.4 months, hazard ratio 0.40) and overall survival (15.3 vs. 11.3 months, hazard ratio 0.62) for PSMA RLT in addition to best standard of care versus best standard of care alone [54]. In the phase II TheraP trial, PSMA RLT was associated with a higher PSA response rate, longer progression-free survival, and fewer grade 3 or 4 adverse events when compared with cabazitaxel [55]. Both studies selected patients based on sufficient PSMA expression by PSMA-ligand PET/CT at baseline. Patients who do not meet the VISION PET inclusion criteria, specified in the section on assessment of PSMA expression prior to PSMA-directed RLT, have a poor outcome after PSMA RLT [56]. [^68^ Ga]Ga-PSMA-11 PET was offered for baseline assessment in the VISION study.

The predictive value of PSMA-ligand PET/CT for survival following the initiation of PSMA RLT was demonstrated in a multicenter study. Among 18 pretherapeutic clinicopathologic and PSMA-ligand PET/CT variables, six were independently associated with the overall survival [57]. Among these, SUV_mean_ of whole-body tumor burden, number of lesions, and the presence of bone or liver metastases were significant survival predictors derived from PET/CT [57]. Short survival associated with low PSMA expression or the presence of liver metastases on PSMA-ligand PET/CT has been confirmed by several studies of PSMA RLT, including trials with additional [^18^F]F-FDG PET for disease localization [58–61].

## Potential clinical applications

### Guidance of prostate biopsy

PSMA-ligand PET/CT improves tumor localization and guides repeated biopsies in patients with high suspicion of prostate cancer and prior negative biopsies [62–64]. In the prospective PRIMARY study, the addition of [^68^ Ga]Ga-PSMA-11 PET to multiparametric MRI significantly improved the negative predictive value (91% vs. 72%, *p* < 0.001) and the sensitivity (97% vs. 83%, *p* < 0.001) for clinically significant prostate cancer [65]. PSMA-ligand PET should be combined with multiparametric MRI for biopsy guidance. MRI delivers anatomic information for fusion biopsy and improves diagnostic confidence by additional lesion information from multiparametric acquisition [62].

### Imaging metastatic prostate cancer

Imaging assessment of metastatic prostate cancer typically includes bone scan, e.g., using [^99m^Tc]Tc-MDP or -DPD, for osseous metastases and CT or MRI for nodal, soft tissue, and visceral metastases. Several studies have demonstrated high diagnostic performance of PSMA-ligand PET/CT for the staging of advanced prostate cancer [66, 67]. The diagnostic accuracy of PSMA-ligand PET/CT for bone assessment was superior to that of bone scan [68, 69]. When compared with conventional imaging, the superior accuracy of PSMA-ligand PET/CT allows for accurate identification of PCWG3 clinical trial target populations, especially in subgroups with nmCRPC or visceral metastatic disease [53]. In patients with oligometastatic disease, PSMA-PET-guided metastasis-directed treatment was associated with high rates of treatment response [70–72].

While PSMA-ligand PET/CT may be an emerging staging tool for metastatic prostate cancer, its impact on management and patient outcome has not yet been sufficiently assessed.

### Monitoring of systemic treatment in metastatic prostate cancer

Despite the proven superiority of PSMA-ligand PET for prostate cancer staging, its role in monitoring treatment response remains less clear. In metastatic prostate cancer, treatment response is currently evaluated using conventional imaging (CT and bone scan) according to the Prostate Cancer Working Group Criteria 3 (PCWG3) guidelines [46]. Several studies assessing different imaging readouts demonstrate the value of PSMA-ligand PET for the assessment of prostate cancer response [73–79]. Recently, the PSMA PET Progression (PPP) criteria [80] and the Response Evaluation Criteria In PSMA-imaging (RECIP) 1.0 [79] were proposed for standardized response assessment. PPP criteria were formed by expert recommendation, whereas RECIP criteria were additionally validated by overall survival in a multicenter cohort of patients undergoing ^177^Lu-PSMA RLT [79].

## Implementation in clinical guidelines

Recommendations for prostate cancer staging in national and international clinical guidelines are under evaluation. PSMA-ligand PET/CT was included in various clinical guidelines and consensus documents for imaging primary disease, BCR, BCP, or metastatic prostate cancer. Recommendations were made following different guideline formats. Therefore, the wording of statements on the role of PSMA-ligand PET/CT are cited directly from the respective document text and summarized in Table 2. In the interest of brevity, Table 2 does not present full statements or complete summaries of all available national and international guidelines. For full statements, background, strength of recommendations, or underlying evidence, we refer to the respective clinical guideline/consensus document.

**Table 2:** Wording of clinical guidelines on the value of PSMA-ligand PET/CT for primary, biochemical persistence (BCP), biochemical recurrence (BCR), and metastatic prostate cancer assessments

Document led by	Initial staging	Localization of BCP	Localization of BCR	Metastatic*	Reference
EAU	“more accurate”	“offer”	“perform”	N/A*	[45, 47, 48]
ESMO	“better sensitivity and specificity than CT or bone scan”	N/A	“replacing conventional imaging”	N/A*	[81]
ASCO	“consider”	“should be offered”	“should be offered”	N/A*	[82]
NCCN	“equally effective, if not more effective” compared to conventional imaging”	“equally effective, if not more effective” compared to conventional imaging”	“equally effective, if not more effective” compared to conventional imaging”	N/A*	[83]

*N/A*, not evaluated. *PSMA-ligand PET/CT is required before PSMA-directed RLT

Currently, several guidelines highlight the superior accuracy of PSMA-ligand PET for the staging of primary disease (EAU, ESMO, NCCN) or consider additional value (ASCO) in this setting. PSMA-ligand PET/CT evaluation of BCR/BCP is recommended in documents produced by the EAU, ASCO, and NCCN. All documents summarized in Table 2 recommend PSMA-ligand PET/CT for the localization of BCR or state superiority over conventional imaging in this setting. No recommendations were made for the assessment of advanced or metastatic prostate cancer outside pre-RLT staging.

## Qualifications and responsibilities of personnel

See the EANM procedure guidelines for tumor PET imaging version 2.0 or the SNMMI Procedure Standard for General Imaging [10, 84].

## Procedure/specification of the examination

### Necessary data for requesting PSMA-ligand PET/CT

As reported previously [11], a request for PSMA-ligand PET/CT should be accompanied by a concise summary of the patient’s history with a focus on diagnosis, risk group, and oncological history. Aspects that should be considered in the review of the patient’s files are given in the following list:

1. Indication for the imaging study
2. Prostate-cancer-specific history:
  - Primary prostate cancer
    i. PSA and Gleason score
    ii. Prior local intervention/biopsy
  - Biochemical recurrence: PSA and PSA kinetics (if available)
  - Current or prior prostate cancer treatments with dates: ADT or other AR-targeted treatments. Prior history of AR-targeted treatment, chemotherapy, radium-223, PSMA-targeted therapy, prostatectomy/surgery/biopsy, and/or radiation therapy
  - Relevant symptoms (e.g., bone pain, frequent urination, nocturia, hematuria, dysuria, impotence, erectile dysfunction, or painful ejaculation)
  - Previous imaging findings including previous PSMA-ligand PET and tracer subtype if known
3. Relevant co-morbidities:
  - Non-prostate malignancies
  - Allergies
  - Renal failure

#### Patient preparation

Patients do not need to fast and may take all their medications. New onset of ADT was associated with decreased PSMA-ligand uptake on PET in patients with hormone-naïve or hormone-sensitive cancer, possibly due to effective tumor reduction [85, 86]. Therefore, PSMA-ligand PET/CT should be performed before the onset of new ADT whenever possible. The influence of second-line androgen modulation in patients with the castration-resistant disease has not been clearly defined yet. Signaling pathways and the temporal impact of androgen modulation on clinical PSMA-ligand PET/CT performance require further study.

Patients should be encouraged to drink a sufficient amount of water to ensure adequate hydration before the PET study. In some circumstances, high residual activity in the urinary system may lead to so-called halo-artefacts in PET. For PSMA-ligands with kidney-dominant excretion (Table 3), activity in the ureters and bladder might lead to false positive or negative findings. Furosemide administration (20 mg i.v., shortly before or after administration of PSMA-ligands) may be especially useful in these situations. Furosemide should not be administered in patients with medical contraindications including urinary incontinence, urinary obstruction, and hypersensitivity to furosemide. Alternatively, oral hyperhydration (1L) during the uptake time followed by bladder voiding immediately before image acquisition can be considered in patients with adequate bladder control.

**Table 3:** PSMA-ligands for PET/CT imaging

Characteristic	[^68^ Ga]Ga-PSMA-11	[^68^ Ga]Ga-PSMA-I&T	[^18^F]F-DCFPyL	[^18^F]F-PSMA-1007	[^18^F]F-rhPSMA-7.3
Binding motif	Urea-based	Urea-based	Urea-based	Urea-based	Urea-based
Half-life	68 min	68 min	110 min	110 min	110 min
Dominant excretion route	Kidney	Kidney	Kidney	Liver	Kidney
Published	2012 [15]	2015 [18, 94]	2011 [14], 2015 [19]	2016 [20]	2020 [21]
Status	Extensive retrospective data; completed phase 2/3; Approved*	Extensive retrospective data	Extensive retrospective data; completed phase 2/3; Approved*	Extensive retrospective data; completed phase 2/3; Approved*	Extensive retrospective data; under phase 3 investigation (NCT04186819 and NCT04186845)

Characteristics and current status. *Refers to regulatory approval for clinical use and distribution on a national or international level

#### Hyperthyroidism and kidney failure

As reported previously [11], PSMA-ligand PET/CT can be performed in patients with hyperthyroidism and kidney failure. However, if intravenous iodinated CT contrast is being considered for the CT protocol, thyroid and renal function should be considered. For details, we refer to the European Society of Urogenital Radiology Contrast Media Guidelines in Europe [87] and to the American College of Radiology Manual on Contrast Media in the USA [88].

#### Radiopharmaceuticals

Several ^68^ Ga- and/or ^18^F-labelled ligands have been developed and assessed in clinical trials [19–21, 26, 89–93]. The majority of current ligands in use are based on a urea-like binding motif and were designed for intravenous administration. Table 3 summarizes PSMA-ligands that have been reported in the literature and are most advanced in the process of clinical implementation and/or approval.

PSMA-ligand PET/CT is performed using an approved product, within the confines of a research study, or based on regulations for non-approved radiopharmaceuticals, respectively. Due to ongoing development, a non-complete overview of the current radioligand availability is summarized here.

[^68^ Ga]Ga-PSMA-11, [^18^F]F-DCFPyL, and [^18^F]F-PSMA-1007 were assessed in phase II/III prospective clinical trials. Several new drug applications for [^68^ Ga]Ga-PSMA-11 and [^18^F]F-DCFPyL were approved by the United States Food and Drug Administration in 2020 [95], 2021 [96, 97], and 2022 [98]. Since the start of 2021, a [^68^ Ga]Ga-PSMA-11 radiolabelling kit has been approved for clinical use by the Australian Therapeutic Goods Administration (TGA) [99]. [^18^F]F-PSMA-1007 recently received regulatory approval for clinical use in France [100]. Furthermore, multiple European institutions hold local manufacturing licenses for ^68^ Ga- and ^18^F-based PSMA-ligands and [^68^ Ga]Ga-PSMA-11 radiolabelling kits are available in several European countries. PSMA-ligands should be manufactured under Good Manufacturing Practice (GMP) conditions and quality control should follow the governing pharmacopeia monograph or national regulations, whichever is applicable.

[^68^ Ga]Ga-PSMA-I&T and [^18^F]F-rhPSMA-7.3 have been assessed extensively including published data on dosimetry and diagnostic performance. [^18^F]F-rhPSMA-7.3 is currently under phase III prospective clinical investigation (NCT04186819 and NCT04186845).

The committee further notes that tracer development is ongoing. Several novel low-molecular-weight ligands for human PSMA, including compounds with a different binding motif or radionuclide label for PET or scintigraphy, are under development (NCT04868604, NCT04838626 among others). Moreover, albumin binder conjugates are under evaluation [101].

#### PSMA-ligand application and administered activity

The administration protocol is summarized in Table 4. PSMA-ligands are injected via intravenous bolus. Injected activity and uptake time have been defined in the prescribing information for [^68^ Ga]Ga-PSMA-11, [^18^F]F-DCFPyL, and [^18^F]F-PSMA-1007, and in clinical trial protocols for [^18^F]F-rhPSMA-7.3 (NCT04186819 and NCT04186845). For ^68^ Ga-labelled ligands, variation in injected activity and volume may be caused by the short half-life of ^68^ Ga and variable elution efficiencies obtained during the lifetime of the ^68^Ge/^68^ Ga radionuclide generator. Cyclotron-produced gallium may help alleviate the issues related to the low output of the ^68^Ge/^68^ Ga radionuclide generator [102]. To maximize the use of the dispensed activity, the administration syringe should be flushed with at least the same volume of saline (NaCl 0.9%). Then, subsequent emptying into the intravenous access is recommended.

**Table 4:** Patient preparation and PSMA-ligand administration

Item	[^68^ Ga]Ga-PSMA-11	[^68^ Ga]Ga-PSMA-I&T	[^18^F]F-DCFPyL	[^18^F]F-PSMA-1007	[^18^F]F-rhPSMA-7.3
Activity	111–259 MBq (3–7 mCi)	111–259 MBq (3–7 mCi)	296–370 MBq (8–10 mCi)	210–280 MBq (3–4 MBq/kg body mass)	296 MBq (8 mCi)
Uptake time	60 min (acceptable range: 50 to 100 min)	60 min (acceptable range: 50 to 100 min)	60 min	90–120 min	60 min
Consider hydration* and/or furosemide (20 mg intravenous)	Yes	Yes	Yes	No	No

*e.g., oral intake of 1 L of water 1 h prior to acquisition

#### Uptake time

Recommended uptake time is around 60 min for most radioligands (Table 4). The interval between PSMA-ligand injection and imaging should be recorded. If the acquisition leads to indeterminate findings, a late scan, beyond 120 min, may be considered. Late scans may aid in the identification of lesions located near the ureter or the bladder [16].

#### PET/CT acquisition protocol

In accordance with [10], the patient should be positioned supine with both arms elevated above the head, as tolerated by the patient. In this position, beam-hardening artefacts in the abdominal and pelvic regions as well as artefacts caused by truncation of the measured field of view can be avoided. In case PET/CT data are used for radiation therapy planning, the examination should be performed in the exact radiotherapy position. Additionally, the same radiotherapy positioning devices should be used whenever feasible (e.g., indexed table top, laser alignment, and immobilization procedures).

The CT scan should be performed from the vertex to mid-thigh, followed by the PET acquisition (described below). CT acquisition parameters (e.g., kV, mAs, pitch in helical CT, and dose modulation) should be in accordance with institutional protocols. The CT protocol may be modified according to clinical requirements. For instance, the skull should be included in patients with known metastatic disease. In the case of focal symptoms or disseminated disease, coverage may be extended to include the respective body part. Diagnostic CT may be acquired with contrast enhancement for morphologic bone and organ assessments. Additional acquisitions (e.g., deep inspiration chest CT) may be performed. If intravenous CT contrast is used, contrast-enhanced CT in the portal venous phase is recommended.

PET acquisition should start from the mid-thigh and extend to the vertex to exploit reduced PSMA-ligand uptake in the urinary system after pre-scan voiding. Acquisition should proceed from the lower end of the axial field of view cranially to minimize misalignment of the urinary bladder, which tends to fill up during the time of the examination in patients with hydration procedures. PET scans are typically acquired in 3D mode with an acquisition time of usually 1–4 min per bed position (or equivalent speed using continuous table movement) adjusted to the injected activity [103]. Overall, PET coverage should be identical to the anatomical CT scan range.

#### PET/CT image reconstruction

In accordance with our previous guidance [11], image acquisition should be performed in 3D mode with appropriate data corrections (attenuation correction, scatter correction, correction for random coincidences). The diagnostic CT scan may be used for attenuation correction. PET reconstruction should be performed with and without attenuation correction to identify potential reconstruction artefacts caused by the correction algorithm [10]. Reconstructed images should be labelled accordingly (e.g., PET AC, PET NAC, and CT CE) and stored in the local picture archiving and communication system. An example of a PSMA-ligand PET/CT protocol is given in Table 5.

**Table 5:** Example protocol for PSMA-ligand PET/CT image acquisition and reconstruction. *FOV*, field of view

Patient position	Arms elevated above the head, supine
CT protocol	FOV: vertex to mid-thigh; optional contrast phase: portal venous
PET protocol	FOV and acquisition: start from mid-thigh to vertex
PET reconstruction	Ordered-subsets expectation maximization, point-spread function or equivalent; attenuation correction from CT data

### Definitions of volumes of interest

SUV can be normalized to body mass, lean body mass, or body surface area. Thus, SUV measurements may change significantly between different modes of normalization. Therefore, the same mode should be used for serial examinations. The definition of maximum SUV (SUV_max_) was given above. SUV_max_ measurement is recommended to determine tracer uptake in key lesions. Repeat quality control procedures are critical to minimize SUV measurement errors and to maintain high image quality.

## Quality control and inter-institution performance harmonization

Clinical interpretation of PSMA-ligand PET/CT is based on visual analysis. Semi-quantitative SUV can be measured and documented for selected lesions. Reproducibility and image quality are of critical importance, especially for communication between different centers. A consistent PET/CT scanner quality control program contributes to the minimization of measurement errors and helps maintain high image quality.

Quality assurance should include (a) daily quality control and calibration measurements of both the PET and CT components of the imaging system as previously described in the EANM Procedure guidelines for [^18^F]F-FDG tumor imaging [10] and (b) cross-calibration of the PET/CT system. Procedures for calibration and cross-calibration have been published for both ^18^F-based [104, 105] and ^68^ Ga-based [106] PET/CT. Guidance is also provided by the PET/CT manufacturer, UPICT oncology [^18^F]F-FDG PET/CT protocol [107], and EANM Research Ltd. (EARL, Vienna, Austria) accreditation frameworks.

## Normal uptake

**Fig. 1:**
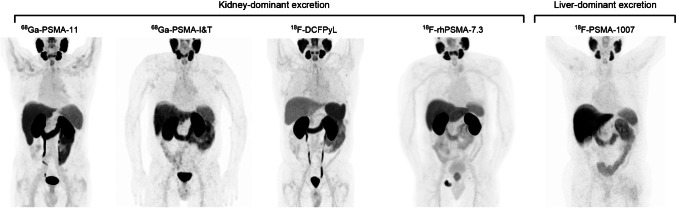
Normal body distribution of PSMA-ligands. [^68^ Ga]Ga-PSMA-11, [^68^ Ga]Ga-PSMA-I&T, [^18^F]F-DCFPyL, and [^18^F]F-rhPSMA-7.3 applications lead to notable kidney uptake. Bladder retention is high for [^68^ Ga]Ga-PSMA-11, [^68^ Ga]Ga-PSMA-I&T, and [^18^F]F-DCFPyL and lower for [^18^F]F-rhPSMA-7.3. Reference organs for ligands with kidney-dominant excretion are liver and parotid gland. [^18^F]F-PSMA-1007 leads to high liver uptake due to hepatic excretion. Reference organs for ligands with liver excretion are spleen and parotid gland. Focal uptake in the pelvic bone is noted on the [^18^F]F-rhPSMA-7.3 PET corresponding to metastatic disease. [^68^ Ga]Ga-PSMA-I&T subpart was modified with permission from [130]

As reported previously [11], normal and variable PSMA-ligand uptake can be found in the following tissues: lacrimal gland, salivary glands, liver, gall bladder, spleen, small intestine, colon, and kidney (Fig. 1).

Usually, tumor lesions inside and outside the prostate gland show a high tumor-to-background ratio compared with the surrounding tissue [16, 62]. [^68^ Ga]Ga-PSMA-11, [^68^ Ga]Ga-PSMA-I&T, and [^18^F]F-DCFPyL are excreted primarily via the urinary system and collected in the bladder; a small proportion is cleared through the hepatobiliary system. Thus, small local recurrences might be missed if the SUV-threshold to judge the PSMA-ligand uptake in soft tissue structures near the urinary bladder is not adjusted properly. Hydration and/or the application of furosemide and/or repeat late acquisition may be useful in such cases.

[^18^F]F-PSMA-1007 shows higher liver and gall bladder accumulation due to hepatobiliary excretion and no or only minimal excretion via the urinary system [108]. Liver uptake is also higher with [^18^F]F-rhPSMA-7.3 than with ^68^ Ga-PSMA-11 and excretion is mainly via the urinary tract. However, retention in the urinary system is usually low at the time of imaging and can be further lowered by application of furosemide [21, 109].

Approximately 5% of all prostate cancers, especially neuroendocrine types, do not exhibit significant PSMA overexpression [110, 111]. Due to physiologic organ uptake, liver metastases with low PSMA expression can be obscured. As neuroendocrine liver metastases often lose PSMA expression, cross-sectional imaging is important for the liver assessment [112–114].

## Important pitfalls

A large number of case reports present imaging findings in PSMA-ligand PET not associated with prostate cancer. Different reviews outline the most important pitfalls and try to give evidence on their biological bases [115–117]. Immunohistochemical and PSMA-ligand PET data have shown that increased PSMA expression can also be found in the neovasculature of non-prostate solid tumors or in benign processes [1, 118–122]. Readers should therefore carefully assess the possibility of a PSMA avid second malignancy. An important pitfall is PSMA-ligand uptake in sympathetic ganglia. Pronounced tracer accumulation can be found for example in the celiac ganglia, which are prone to misinterpretation as retroperitoneal lymph node metastases [123]. For ^18^F-labelled PSMA-ligands, visually recognizable uptake is also reported for other ganglia, especially in the sacral and cervical regions [24, 124, 125]. Ganglia can be differentiated from lymph node metastases by location (adjacent to neuroforamina) or shape (often linear or comma-shaped) [126].

Using the ^18^F-labelled compounds [^18^F]F-PSMA-1007 and [^18^F]F-rhPSMA-7.3, interpretation of bone lesions is more challenging compared to [^68^ Ga]Ga-PSMA-11 [24, 125, 127, 128]. A number of benign bone lesions accumulate PSMA and result in false positives on PSMA-PET/CT, including fractures, osteophytes, benign bone lesions (fibrous dysplasia, hemangioma), or unknown etiology. In the literature, clinically insignificant bone uptake was reported as unspecific bone uptake (UBU, [128]) or non-specific bone lesions (NSBL, [127]), and the nature of these lesions was mainly assessed by clinical follow-up with histological verification performed in few cases. Characteristic CT or MRI findings of benign lesions can help interpretation and comparison to any available previous studies should be performed. [^18^F]F-DCPyL and [^68^ Ga]Ga-PSMA11 demonstrated lower rates of equivocal skeletal findings in separate matched-pair comparisons with [^18^F]F-PSMA1007 [24, 129]. Typical locations for PSMA-avid benign bone lesions are the ribs and pelvis and the intensity of tracer uptake is generally lower than for bone metastases. However, definite discrimination by quantitative measurement is not possible. In the case of single lesions (especially in the ribs) and the absence of a definite morphological correlate typical for malignancy, interpretation of metastasis should be cautious to avoid over-staging. Consequent application of Prostate Cancer Molecular Imaging Standardized Evaluation (PROMISE) criteria for image interpretation can help to avoid false positives [130].

AR inhibition can lead to elevated PSMA expression in prostate cancer lesions [131, 132]. However, extent and timing of upregulation are not completely understood. Time interval between AR inhibition and PET/CT must be considered to prevent false diagnosis of tumor progression after initiation of AR-targeted therapy. The increase in PSMA-ligand uptake might be transient and is most pronounced during the first weeks of ADT with subsequent decline over time [85].

## Complementary information

Comparison with previous examinations should be part of each PSMA-targeted PET report. Assessment is more valuable if the examination is interpreted in the context of other imaging examinations (bone scan, CT, PET/CT, MRI, etc.) and clinical data.

## Documentation and reporting

### Study identification

The final report includes the name and date of birth of the patient, medical record number, and date of the examination.

### Clinical information

Clinical summary includes the diagnosis, a brief history of prior treatments, and the reason for referral with specific question to be answered. In addition, previous adequate diagnostic tests, including PSA level and prior imaging findings, should be summarized. If the study is being done to assess treatment response, details of the most recent treatment regime (including start/stop dates and agent) should be provided. Date and type of comparison studies should be reported. A statement should be made in case no comparison studies are available.

### Technical details

As recommended previously [10], study-specific information should include the radiopharmaceutical, the amount of injected activity in megabecquerels (MBq) and/or millicuries (mCi), the route (intravenous) and anatomical site of administration, the date and time of administration, and the time of any furosemide injection. The time interval between the administration of the PSMA-ligand and the start time of the acquisition should be reported. The part of the body that was covered should be described from the start to the endpoint. The position of the patient (supine or prone) and the position of the arms (elevated or by the sides) should be stated if non-standard.

In case a low-mAs CT was performed, description of the CT part may be limited to attenuation correction and anatomical registration of the emission images. If the CT examination was optimized for diagnosis, then more details should be provided. Dosimetry parameters should be included as required by local regulations. The report should state if contrast agent was given as part of the CT protocol.

Quality issues of the PSMA-ligand PET/CT study should be reported, for example, motion artefacts, potential halo-artefacts due to high activity in the collecting urinary system or the bladder, CT-related artefacts (from radiation attenuating matter/materials, e.g., metals, especially hip prostheses which generate beam hardening and affect pelvic visualization) should be mentioned [10].

### Description of the location, extent, and intensity of PSMA-ligand uptake

In the general review, attention should be paid to the prostate gland/bed, seminal vesicles, vas deferens, regional and distant lymph nodes, bones, lungs, and liver. Regions that may relate to any symptoms or pathology noted on the referral form should be given specific attention. PSMA-ligand accumulation should be reported as absent, low, intermediate, or high by comparison to the background uptake [133] and semi-quantitative values may be reported. Deviations from the physiological tracer distribution should be described, particularly in the kidneys, where clinically relevant renal dysfunction/pathology may be unveiled. PSMA-ligand uptake in incidental findings not related to prostate cancer, such as synchronous malignancies, should also be reported. Tumor lesions usually appear as focal tracer uptake higher than the adjacent background. Frameworks for standardized reporting of PSMA-ligand PET/CT have been developed (see below).

### Standardized reporting

Standardized reporting is increasingly applied for diagnostic procedures [134]. To date, a number of these systems have been developed to assess lesions in specific organs (e.g., breast, liver, thyroid, and prostate). These classifications are usually based on a 5-point (Likert) scale that concords with the probability of a lesion being benign or malignant. In the context of PSMA-ligand PET/CT, a number of frameworks for standardized reporting have been proposed and will undergo modifications over time. Current frameworks are summarized in the following sections.

#### EANM Delphi consensus

In 2017, Fanti et al. [135] published the first effort towards a standardized interpretive approach to PSMA-ligand PET. Seven different readers each provided interpretations of the [^68^ Ga]Ga-PSMA-11 PET/CT scans from 49 patients with BCR. Multiple rounds of Delphi consensus were performed until the final agreement was reached. Those final agreements were used as a basis for consensus guidelines on the interpretation of [^68^ Ga]Ga-PSMA-11 PET/CT. The guidelines included (1) that all sites of unexpected increased radiotracer uptake should be reported as “anomalous,” (2) that any anomalous findings should be categorized as “pathologic” if they are suggestive of prostate cancer, and (3) a series of additional and general recommendations for aspects of the final report.

#### PSMA reporting and data system (PSMA-RADS)

PSMA-RADS proposed in 2018 falls under the umbrella of MI-RADS, a generalizable framework for the interpretation of PET scans utilizing the targeted theranostic radiotracers [136]. This reporting system follows the basic structure of other RADS approaches, such as the Breast Imaging Reporting and Data System (BI-RADS) or the Prostate Imaging Reporting and Data System (PI-RADS) [136]. Its goal is to convey the imaging specialist’s level of confidence regarding the presence of prostate cancer at both the individual lesion and the scan level, and to offer recommendations regarding the potential need for any additional work-up. PSMA-RADS includes diagnostic criteria for a series of categories (1, 2, 3, 4, or 5) as well as subcategories (1A, 1B, 3A, 3B, 3C, and 3D). These categories represent an increasing likelihood of the presence of prostate cancer, with PSMA-RADS-1 indicating definitively benign findings and PSMA-RADS-5 indicating the definitive presence of prostate cancer. The indeterminate nature of PSMA-RADS-3 lesions has been validated [137] and the system has high inter-reader agreement [138].

#### Prostate Cancer Molecular Imaging Standardized Evaluation

Also, in 2018, the PROMISE system was proposed as a standardized framework for the evaluation of the PSMA-ligand PET [130]. It defines molecular imaging TNM (miTNM) regions and subregions for whole-body staging, similar to the pathological/clinical TNM system. PROMISE organizes findings in comprehensible categories to report the location of prostate cancer throughout the body including disease distribution pattern and PSMA expression score. The local tumor is described from miT0 (i.e., absence of local recurrence following local therapy) to miT2 through miT4 for tumoral extent in individuals with intact prostates. Pelvic nodal involvement is categorized as miN1 or miN2 depending on the number of pelvic nodal regions involved. Lastly, extrapelvic metastases are indicated by miM1a, miM1b, or miM1c depending on whether extrapelvic nodes, bone, or viscera are involved, respectively. miM1b is further divided into unifocal, oligometastatic, disseminated, or bone marrow carcinomatosis.

#### E-PSMA

Supported by the EANM, an evolution of the earlier Delphi consensus document was developed by a panel of worldwide experts who provided consensus statements for standardized reporting of the PSMA-ligand PET [139]. Panelists were selected based on their expertise and publication record in the diagnosis or treatment of prostate cancer, their involvement in clinical guidelines, and according to their expertise in the clinical use of PSMA-ligands. Statements were formed as part of a Delphi consensus process. E-PSMA provides an overview of the experts’ opinion regarding what needs to be included in a report, what different systems for reporting exist, and what is important to report in different clinical settings. Finally, the panelists’ recommendations were summarized in a structured report for PSMA-ligand PET including elements from the PROMISE, miTNM, and RADS systems [130, 136].

#### The PRIMARY score for prostate cancer diagnosis

Emmett et al. assessed patterns of intra-prostatic PSMA and proposed a 5-point PRIMARY score for PSMA-ligand PET/CT detection of prostate cancer. In a prospective multicenter phase II study, the PRIMARY score identified clinically significant prostate cancer with high accuracy and inter-reader agreement [140].

#### Assessment of PSMA expression prior to PSMA-directed RLT

To evaluate eligibility for PSMA-targeted RLT, the following information should be reported: (1) overall visual uptake intensity of prostate cancer lesions in reference to liver ([^68^ Ga]Ga-PSMA-11, [^68^ Ga]Ga-PSMA-I&T, [^18^F]F-DCFPyL, [^18^F]F-rhPSMA-7.3) or spleen ([^18^F]F-PSMA-1007). Uptake greater than that of the reference organ parenchyma will be regarded as positive. Uptake equal to or lower than that of the reference organ in any lymph node with a short axis of at least 2.5 cm or any metastatic soft tissue lesion with a short axis of at least 1.0 cm (for organ and bone with soft tissue component) will be regarded as negative, in accordance with VISION criteria [141]. The location and extent of PSMA-negative lesions should also be reported. Information on prostate cancer SUV and number of lesions provides additional prognostic information [57].

#### Assessment of response to therapy

Two frameworks were proposed for the assessment of response, although there are limitations to the use of these frameworks for hormone-based therapies. PPP criteria were proposed based on expert recommendations [80]. PPP criteria include assessment of biochemical or clinical progression along with PSMA-ligand PET lesion count.

The Response Evaluation Criteria In PSMA-imaging (RECIP) were proposed to evaluate treatment efficacy using PSMA-ligand PET in metastatic castration-resistant prostate cancer patients [79]. The RECIP design is based on findings from a multicenter analysis of RLT outcomes. PET was performed at baseline and 12 weeks after RLT initiation. In a head-to-head comparison, RECIP achieved highest diagnostic value and inter-reader reliability when compared to adapted PCWG3, RECIST, PERCIST, and PPP criteria [142]. Whereas PPP relies on the appearance of new lesions or biochemical or clinical progression, RECIP assesses new lesions along with changes in total PSMA tumor volume. Both frameworks were recently proposed and may need additional validation before widespread implementation. A summary of the PPP and RECIP criteria is presented in Table 6.

**Table 6:** Summary of the PPP and RECIP criteria for PSMA-ligand PET/CT-based response assessment

Criteria	Definition
*PPP *[80]	
Progressive disease	(a) Appearance of ≥ 2 new PSMA-positive distant lesions or (b) Appearance of 1 new PSMA-positive distant lesion plus consistent clinical and/or laboratory data (including changes in serum PSA, lactate dehydrogenase, alkaline phosphatase levels, or ECOG score) or (c) Increase in size or PSMA uptake of ≥ 1 existing lesions by 30% plus consistent clinical and/or laboratory data
*RECIP 1.0* [79]	
Complete response	Absence of any PSMA uptake on follow-up PET scan
Partial response	≥ 30% decrease in PSMA-VOL without appearance of new lesions
Progressive disease	≥ 20% increase in PSMA-VOL with appearance of new lesions
Stable disease	Does not meet the above criteria

*PSMA-VOL*, PSMA-ligand PET derived tumor volume

It should be noted that assessments of disease progression at early time points following the initiation of androgen-axis-targeted agents can be difficult because the upregulation of PSMA as a result of the interruption of androgen signaling may change the tracer uptake and the apparent extent of the disease [143, 144]. As a result, currently proposed response assessment criteria may be of greater value when used at later times of a given systemic therapeutic approach [145].

### Summary and diagnosis/impression

The overall scan interpretation of PSMA-ligand PET studies must be clearly reported as normal or abnormal. A qualitative estimate of the likelihood of a diagnosis and the differential diagnoses should be given. Questions in the study referral should be addressed directly [10]. Report summaries should be structured to the main tumor sites (local tumor involvement, lymph node, or bone metastases) and potential other lesions. Standardized reporting should be applied for disease location and certainty of diagnosis [130, 135, 136, 139].

## Radiation exposure to the patient

Radiation exposure from the radiopharmaceutical (Table 7) and the CT study contribute to the total radiation dose with PSMA-ligand PET/CT. The mean dose for a CT scan is variable and depends on the protocol and CT hardware. Recent advances have led to significant radiation dose reduction attributable to the CT component.

**Table 7:** Radiation dosimetry for PSMA-ligands

		[^68^ Ga]Ga-PSMA-11	[^68^ Ga]Ga-PSMA-I&T	[^18^F]F-DCFPyL	[^18^F]F-PSMA-1007	[^18^F]F-rhPSMA-7.3
Reference		[146]	[18]	[147]	[108]	[148]
Effective dose coefficient	mSv/MBq	0.0169	0.0199	0.0116	0.022	0.014
Urinary bladder wall	mGy/MBq	0.0982	0.0674	0.0072	0.0187	0.012
Kidneys	mGy/MBq	0.3714	0.22	0.123	0.170	0.172

Based on the available studies (Table 7), the coefficient for effective dose from PSMA-ligand application ranges from 0.0116 to 0.022 mSv/MBq resulting in an average effective radiation dose of 3.4/4.0 mSv for 200 MBq [^68^ Ga]Ga-PSMA-11/[^68^ Ga]Ga-PSMA-I&T, or 3.5/6.6/4.2 mSv for 300 MBq [^18^F]F-DCFPyL/[^18^F]F-PSMA-1007/[^18^F]F-rhPSMA-7.3. The radiation exposure related to a CT scan carried out as part of a PSMA-ligand PET/CT study depends on the intended use of the CT. The effective dose ranges from 1 to 20 mSv for the CT part depending on the protocol (low-dose CT and/or diagnostic CT). Given the variety of CT hardware and protocols, the radiation exposure for a PSMA-ligand PET/CT study should be calculated specifically for a given protocol.

## Liability statement

This guideline summarizes the views of the EANM Oncology & Theranostics Committee and SNMMI. It reflects recommendations for which the EANM/SNMMI cannot be held responsible. The recommendations should be taken into context of good practice of nuclear medicine and do not substitute for national and international legal or regulatory provisions.
